# Intra‐gastrointestinal amyloid‐β1–42 oligomers perturb enteric function and induce Alzheimer's disease pathology

**DOI:** 10.1113/JP279919

**Published:** 2020-07-06

**Authors:** Yayi Sun, Nerina R. Sommerville, Julia Yuen Hang Liu, Man Piu Ngan, Daniel Poon, Eugene D. Ponomarev, Zengbing Lu, Jeng S. C. Kung, John A. Rudd

**Affiliations:** ^1^ School of Biomedical Sciences; ^2^ Faculty of Medicine the Laboratory Animal Services Centre The Chinese University of Hong Kong New Territories Hong Kong

**Keywords:** alzheimer's disease, beta‐amyloid, brain‐gut axis

## Abstract

**Key points:**

- Alzheimer's disease (AD) patients and transgenic mice have beta‐amyloid (Aβ) aggregation in the gastrointestinal (GI) tract.
- It is possible that Aβ from the periphery contributes to the load of Aβ in the brain, as Aβ has prion‐like properties.
- The present investigations demonstrate that Aβ injected into the GI tract of ICR mice is internalised into enteric cholinergic neurons; at 1 month, administration of Aβ into the body of the stomach and the proximal colon was observed to partly redistribute to the fundus and jejunum; at 1 year, vagal and cerebral β‐amyloidosis was present, and mice exhibited GI dysfunction and cognitive deficits.
- These data reveal a previously undiscovered mechanism that potentially contributes to the development of AD.

**Abstract:**

Alzheimer's disease (AD) is the most common age‐related cause of dementia, characterised by extracellular beta‐amyloid (Aβ) plaques and intracellular phosphorylated tau tangles in the brain. Aβ deposits have also been observed in the gastrointestinal (GI) tract of AD patients and transgenic mice, with overexpression of amyloid precursor protein. In the present studies, we investigate whether intra‐GI administration of Aβ can potentially induce amyloidosis in the central nervous system (CNS) and AD‐related pathology such as dementia. We micro‐injected Aβ1–42 oligomers (4 μg per site, five sites) or vehicle (saline, 5 μl) into the gastric wall of ICR mice under general anaesthesia. Immunofluorescence staining and *in vivo* imaging showed that HiLyte Fluor 555‐labelled Aβ1–42 had migrated within 3 h via the submucosa to nearby areas and was internalised into cholinergic neurons. At 1 month, HiLyte Fluor 555‐labelled Aβ1–42 in the body of the stomach and proximal colon had partly re‐distributed to the fundus and jejunum. At 1 year, the jejunum showed functional alterations in neuromuscular coupling (*P *< 0.001), and Aβ deposits were present in the vagus and brain, with animals exhibiting cognitive impairments in the Y‐maze spontaneous alteration test (*P *< 0.001) and the novel object recognition test (*P *< 0.001). We found that enteric Aβ oligomers induce an alteration in gastric function, amyloidosis in the CNS, and AD‐like dementia via vagal mechanisms. Our results suggest that Aβ load is likely to occur initially in the GI tract and may translocate to the brain, opening the possibility of new strategies for the early diagnosis and prevention of AD.

## Key points

- Alzheimer's disease (AD) patients and transgenic mice have beta‐amyloid (Aβ) aggregation in the gastrointestinal (GI) tract.
- It is possible that Aβ from the periphery contributes to the load of Aβ in the brain, as Aβ has prion‐like properties.
- The present investigations demonstrate that Aβ injected into the GI tract of ICR mice is internalised into enteric cholinergic neurons; at 1 month, administration of Aβ into the body of the stomach and the proximal colon was observed to partly redistribute to the fundus and jejunum; at 1 year, vagal and cerebral β‐amyloidosis was present, and mice exhibited GI dysfunction and cognitive deficits.
- These data reveal a previously undiscovered mechanism that potentially contributes to the development of AD.

## Introduction

Attempts to prolong life expectancy are currently threatened by an increase in age‐related diseases, especially those involving progressive neurodegenerative diseases resulting in dementia (Niccoli & Partridge 2012). Alzheimer's disease (AD) is the most common age‐related cause of dementia – accounting for approximately 60% to 80% of all cases – and is characterised by beta‐amyloid (Aβ) plaques and intracellular phosphorylated tau tangles in the brain (Washington *et al*. 2016). Even though AD was identified more than a century ago, no effective strategy for treatment or early diagnosis yet exists; current treatment provides only temporary relief of symptoms. Unfortunately, the possibility of discovering new drugs for AD using conventional animal models is low. In the past decade, 99.6% of candidate drugs shown to be effective in animal models failed in human trials (Cummings *et al*. 2014). Therefore, there is an urgent need to explore innovative approaches to prevent AD, such as developing and optimising animal models that have an improved translational power.

Although the exact mechanism of AD pathogenesis is still not fully understood, a wealth of studies indicate that Αβ triggers the degenerative processes in a self‐propagating manner (Eisele & Duyckaerts 2016). Information gained from studies of brain tissues from AD patients show that Αβ deposits occur in the neocortex and hippocampus, followed by the lenticular nucleus, thalamus, mesenphalon and finally the cerebellum (Jarrett & Lansbury Jr. 1993; Jarrett *et al*. 1993). Likewise, Αβ ‘seeds’ inoculated into the brains of animal models can be retrogradely or anterogradely propagated, finally spreading via axonal transport to the overlying cortex and hippocampus to cause cognitive deficits. In the periphery, Αβ deposits have been observed in the GI tract of patients and in transgenic mice that overexpress amyloid precursor protein (APP) (Eisele *et al*. 2007; Eisele & Duyckaerts 2016). The colon is believed to be the first segment of the GI tract where Αβ deposits occur (Hui *et al*. 2012), and it has been hypothesised that Αβ could migrate from the periphery to the brain (Cintron *et al*. 2015). Indirect evidence from experiments involving an intraperitoneal injection of Αβ show an enhanced aggregation of Αβ in the brains of APP23 transgenic mice to support a retrograde transport route. As Αβ appears to share similarities with prion proteins, peripheral Αβ seeds may also be reasonably expected to migrate in a prion‐like manner (Watts *et al*. 2014). Compared with the neuropathological mechanisms in Creutzfeldt–Jakob disease, the possibility exists that cerebral Αβ amyloidosis might originate from sites in the periphery by utilising circulating monocytes as a vector to traverse the blood–brain barrier (Jaunmuktane *et al*. 2015).

Related studies in rats demonstrated that alpha‐synuclein injected into the stomach is transported retrogradely via the vagus to the dorsal motor nucleus of the vagus nerve in the brainstem (Holmqvist *et al*. 2014). It is believed that a failing enteric nervous system contributes to elements of the GI disorders seen in Parkinson's disease (PD) (Poirier *et al*. 2016). However, the effect of Αβ on the GI tract is unknown, nor is it known whether enteric Αβ seeds can be retrogradely transported to the brain and induce long‐term gastric and cognitive deficits. It remains possible that neuronal loss in the GI tract occurs years ahead of the CNS manifestation of AD. If the alterations in function initially occur in the GI tract before the brain, study of enteric neuronal reactivity and neuromuscular coupling could provide insight into a possible early diagnosis of AD while also opening the possibility of a new strategy for early treatment.

## Methods

### Ethical approval

All the procedures and experiments were conducted in accordance with permission from the Animal Experimentation Ethics Committee (reference no. AEEC 15/026/MIS), the Chinese University of Hong Kong, and were conducted under a licence from the Government of the Hong Kong SAR.

### Animals

Two‐month‐old male ICR mice were obtained from the Chinese University of Hong Kong. They were housed at 24 ± 1°C with 50 ± 5% humidity under a 12 h/12 h light/dark cycle. Food and water were provided *ad libitum*. Fifteen and 20 mice were induced in the vehicle and Aβ1–42 treated groups, respectively.

### Formulation of oligomeric Aβ1–42

HiLyte Fluor 555‐labelled Aβ1–42 (AS‐60480‐01, Anaspec, Fremont, CA, USA) was used to track and localise Aβ1–42 from its injection site using a fluorescence *in vivo* imaging system. Non–Fluor‐labelled Aβ1–42 (AS‐20276‐25, Anaspec, Fremont, CA, USA) was utilised in long‐term studies. Solutions of oligomeric Aβ1–42 were prepared as previously described. Briefly, Aβ1–42 peptide was dissolved in saline and sonicated at 37°C for 5 min, then incubated at 4°C for 24 h (Hong *et al*. 2009). The resulting oligomers were verified by atomic force microscopy, according to previous publications (Maezawa *et al*. 2006; Moon *et al*. 2011).

### Surgery

**Figure 1:**
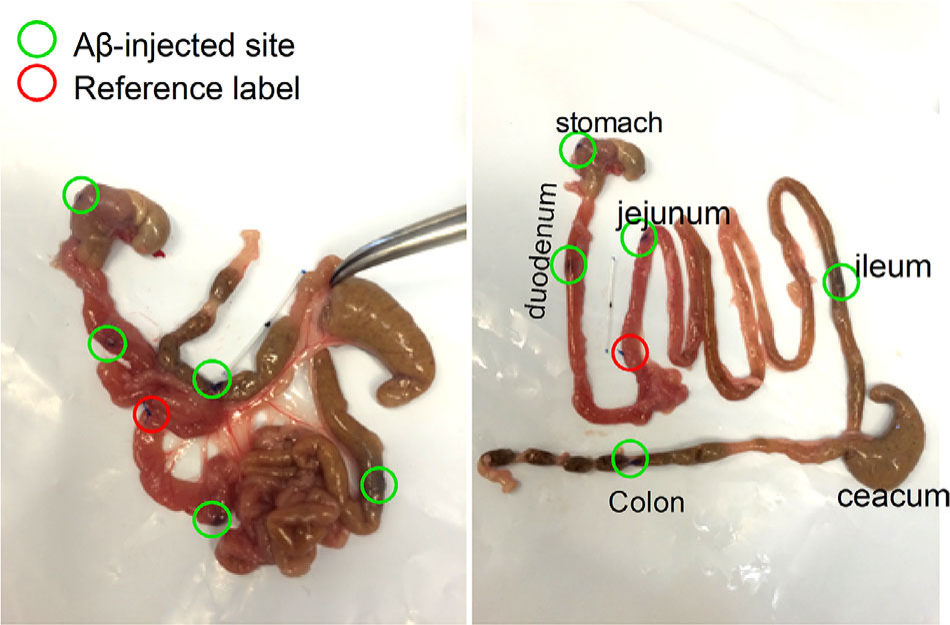
Administration of Aβ through the serosa lining of different regions of the GI tract Five injections are shown for body of stomach, duodenum, jejunum, ileum and proximal colon (indicated by green circles). These sites were located after dissection with the aid of non‐absorbable suture knots (indicated by red circles) and/or specific anatomical structures. [Color figure can be viewed at wileyonlinelibrary.com]

Two‐month‐old mice were anaesthetised with 2.5% isoflurane (Abbott Laboratories, Lake Bluff, IL, USA) in oxygen using a table‐top compact anaesthetic machine (Vetequip Incorporated, Pleasanton, CA, USA). After mice had lost their righting reflex, they were injected with buprenorphine (0.1 mg/kg, s.c.; Schering Plough, NJ, USA) and xylazine (3 mg/kg, i.p.; Alfasan International Ltd., Woerden, Holland). Mice were then placed in a dorsal recumbent position on a heated pad (Vetequip Incorporated, Pleasanton, CA, USA) and maintained at 36°to 37°C. Ocular lubricant (Lubrithal, Dechra, Shropshire, UK) was applied to both eyes to prevent corneal desiccation. They were maintained in the anaesthetised state using 0.2% to 0.8% isoflurane in oxygen. An incision was made in the skin and the abdominal wall. The linea alba was then incised using surgical scissors. Two major experiments were conducted. The first involved the administration of HiLyte Fluor 555‐labelled Aβ1–42 (4 μg, each injection) or vehicle (saline, 5 μl) into the serosa of the body of the stomach and colon, to enable tracking of Aβ by immunofluorescence techniques at 1 month. The second involved the administration of unlabelled Aβ1–42 oligomers or vehicle (saline, 5 μl) into the body of the stomach, duodenum, jejunum, ileum and proximal colon. In both experiments, the injections were made into the serosa, to a depth of 1–2 mm, using a syringe and needle (50 μl gas‐tight syringe, 35‐gauge with point style 4 removable needle; Hamilton Company, Reno, NV, USA). The dose of Aβ at each site was 4 μg; each site of injection was also tagged using non‐absorbable suture knots adjacent to the injection sites, so the target area could be found subsequently by visual inspection (Fig. 1). During the procedure, abdominal contents and GI tissue were moistened regularly using sterile cotton buds and saline. Absorbable suture (6‐0 Vicryl, Polyglactin 910, FS‐3, Ethicon, NJ, USA) was used to close the abdomen using a continuous stitching pattern (Riet‐Correa *et al*. 2002). After closure of the skin, oxygen was provided to the mice to facilitate recovery. During the first week post‐surgery, mice were carefully observed and buprenorphine (0.1 mg/kg, s.c.) was administered daily. Post‐operative complications were rare, accounting for approximately 1% of surgical cases.

### *In vivo* imaging

Alexa Fluor 555‐labelled Aβ oligomers in the whole isolated GI tracts were assayed using a Bruker *In‐Vivo* Xtreme imaging system (Bruker Corporation, Billerica, MA, USA) at four time points (3 h, 3 days, 1 week and 1 month post‐injection). A high‐sensitivity 4MP camera was used to acquire image data on a 2048 × 2048 pixel charged‐coupled device. Single‐frame image data were digitized at 16 bits, using Bruker molecular‐imaging software as a 32‐bit floating point image. A standard Acquire Window was accessed from the software with defined parameters to detect the injected fluorescence‐labelled oligomers.

### Isolation of the myenteric plexus

Mice were killed by CO_2_ asphyxiation, after which their entire GI tracts were removed from the abdominal cavity. Tissues were placed immediately into cold phosphate‐buffered saline and kept on ice during myenteric plexus isolation. The adipose tissues and surrounding blood vessels were removed, and the lumen was rinsed with phosphate‐buffered saline. Separate segments to be studied were pinned to a petri dish with serosa side down. The mucosa layers were removed using scissors, and the remaining tissues were fixed in 4% paraformaldehyde (PFA) for whole‐mount staining (Grundmann *et al*. 2015).

### Organ bath experiments

In some experiments, mice were killed by CO_2_ asphyxiation at 1 year after the intra‐GI administration of unlabelled Aβ1–42 oligomers or vehicle (saline, 5 μl). One‐centimetre segments of the body of the stomach, duodenum, jejunum, ileum and proximal colon were dissected, and the extrinsic blood vessels and nerves along the mesenteric border were trimmed away. The tissues were immediately transferred to Krebs solution (composition in mM: NaCl 118, CaCl_2_ **·**2H_2_O 2.5, KH_2_PO_4_ 1.2, KCl 4.7, MgSO_4_ **·**7H_2_O 1.2, NaHCO_3_ 25 and glucose 10) and suspended under 0.5 to 1 *g* tension (corpus of stomach and proximal colon, 1 *g*; duodenum, jejunum and ileum, 0.5 *g*) in a 10 ml tissue bath containing Krebs solution, bubbled with 5% CO_2_/95% O_2_ at 37°C. After equilibration for 60 min, the spontaneous contractile motility was recorded. Subsequently, electrical field stimulation (EFS) was applied to evaluate the contractility of longitudinal muscle and thus determine the local nerve network‐mediated motor responsiveness. The EFS protocol is briefly described here. The longitudinal segments were stimulated via two parallel platinum electrodes connected to a Grass stimulator. Each segment was subjected to individual frequencies of 2, 4, 8, 16 and 32 Hz at a voltage of 40 V, with 0.2 ms rectangular pulse duration. Three repeated stimuli were delivered for 10 s of train duration, every 1 min, as an inter‐train interval. Changes in muscle tension were recorded using isometric force transductors (ADI Instruments, Chalgrove, UK) using data acquisition Chart software, and contractions were recorded isometrically using Chart software (Chart, version 3.5s/s MacLab, New South Wales, Australia). In addition, the pharmacological characterisation of the responses evoked by EFS were investigated using 1 μM tetrodotoxin (TTX), which was applied 7 min prior to measuring the effects without enteric neuron‐driven responses (Broad *et al*. 2013). Due to the biphasic nature of the responses to EFS (contractions or relaxations during EFS, and an additional contraction after EFS), the effects of EFS were measured by comparing the calibrated wave patterns among different groups over a 40 s duration, from the initial 20 s baseline prior to the stimulation, and then 10 s EFS‐on and subsequent 10 s EFS‐off movement changes. The wave patterns of segments were computed using Microsoft Excel.

### Microelectrode array (MEA)

At 1 year after surgery, mice were killed by CO_2_ asphyxiation and the small intestine was dissected and prepared for *ex vivo* slow‐wave determinations. An MEA system was used to detect the signals as previously described (Nakayama *et al*. 2009). Briefly, 1 cm segments were fixed in a recording chamber, with the longitudinal muscle layer (the serous membrane) directly connected placed on an array of 8 × 8 planar microelectrodes (150 μm in polar distance). The tissue was kept in place by a slice anchor (HARP SLICE GRIDS, ALA Scientific Instruments, Farmingdale, NY, USA). The electrodes were connected to a multichannel amplifier recording system for signal acquisition (a 60‐channel system, Multichannel Systems, Reutlingen, Germany). During the testing session, the recording chamber was perfused with Krebs solution containing 1 μm nifedipine at 37°C. Five‐minute recordings of different segments (duodenum, jejunum and ileum) were made, and data were processed using Spike2 software (Cambridge Electronic Design Ltd., Cambridge, UK) for further statistical analysis.

### Immunofluorescence and immunohistochemistry

One year after surgery, mice were killed by pentobarbitone sodium (80 mg/kg, i.p.) and transcardially perfused with 4% ice‐cold PFA. Tissues were collected in 4% PFA for post‐fixation and transferred into a 30% sucrose solution for 2 days. The tissues were then embedded in O.C.T. compound (Agar Scientific, Stansted, UK) and frozen at ‐80°C. Fifteen micron‐thick coronal sections were cut using a CM 3050C cryostat (Leica ProbeOn Plus charged slides, Leica Biosystems, Wetzlar, Germany) and thaw‐mounted on ProbeOn Plus charged slides (Fisher Scientific, USA). The sections were pretreated with 0.3% hydrogen peroxide for 15 min and then permeabilized using phosphate‐buffered saline solution (containing 5% goat serum and 0.3% Triton X‐100) for another 2 h. Without rinsing, the slides were incubated with anti‐Aβ primary antibody (rabbit, 1:300; ab10148, Abcam, Cambridge, UK) in a cold room overnight, and sequentially incubated with appropriate biotinylated secondary antibody for another 2 h at room temperature. After two rinses, DAB staining was applied for visualisation. Slides were then dehydrated, cleared in xylene and cover‐slipped. The air‐dried slides were then observed using a Q‐imaging digital camera. Both transverse GI sections and myenteric plexuses were stained using anti‐PGP 9.5 (rabbit, 1:500; CL95101, Cedarlane Laboratories, Burlington, ON, Canada) and Anti‐ChAT (goat, 1: 300; sc‐19507, Santa Cruz Biotechnology, Dallas, TX, USA) primary antibodies, together with Alexa‐647 anti‐rabbit (1:500) and FITC anti‐goat (1:300) secondary antibodies, to localise the HiLyte Fluor 555‐labelled Aβ1‐42. As to the vagus nerves, they were double‐stained with anti‐PGP 9.5 and anti‐Aβ (mouse, 1:300; Abcam, Cambridge, UK) primary antibodies, together with Alexa‐647 anti‐rabbit (1:500) and Alexa‐555 anti‐mouse (1:500) secondary antibodies, to identify Aβ deposits among vagal fibres. The mounted tissues were observed using an Olympus FV1000‐ZCD laser scanning confocal microscope.

### Behavioural assessments

The spontaneous Y‐maze, novel object recognition (NOR) and Morris water maze (MWM) tests were conducted 1 year post‐surgery. The spontaneous Y‐maze assessment was used to test short‐term spatial recognition memory. The field consisted of white plastic walls, with each arm 50 cm long, 20 cm high and 10 cm wide at the bottom. Each mouse was placed in the centre of the apparatus and allowed to move freely in the maze. The alteration and total number of arm entries were recorded during 8 min sessions. The maze was cleaned with 70% (v/v) ethanol before each recording session. An alteration was defined as consecutive entries in all three arms (i.e. ABC, CAB or BCA) without repetition (i.e. ABA or ACA), and the percentage of alterations, calculated as [Successive triplet sets (consecutive entry into the three different arms) / Total number of arm entries − 2] × 100%, was compared between different treatment groups. The non‐forced memory test (NOR) task was subsequently conducted. Subjects were transported to an empty open field for habituation, and this lasted 30 min on Day 1; on the following day (Day 2), two identical objects were introduced into the open field, and mice were allowed to explore freely for 10 min as a training (i.e. acquisition) session. After a 24 h stay in their home cages, mice were returned to the same arena, where one of the original objects had been replaced by a new and different object. In this retention session, the amount of time that the mice spent exploring the familiar and novel objects, respectively, was recorded. For statistical analysis, the recognition index was defined as follows: Recognition Index = Time exploring novel object / (Time exploring familiar object + Time exploring novel object) × 100%. Finally, the MWM task was used to evaluate long‐term spatial memory. The test field was a standard circular pool (150 cm diameter) with four different landmarks in orientation; a platform (10 cm diameter) was placed inside the pool. The pool was filled with room‐temperature water to a depth of 45 cm, and the platform was placed 1 cm below the surface. The whole task typically lasted 6 days, with four trials daily, except for the last day. Mice initially had 60 s to freely find the hidden platform. After that they were guided to the target and stayed for another 15 s for memory retention. On the first day, animals were trained using a visible platform, but from the second to the fifth day, they were subjected to a hidden submerged platform. On the last day, the platform was removed, and the tracks of mice were recorded as well. The oriented entrance of each trial was randomly picked without bias or preference. The interval between testing in each of the three tests was 2 days. At the end of the experiments, the mice were killed by CO_2_ asphyxiation.

### Body weight, daily food intake and defecation

Body weight, daily food intake and faecal production (after drying overnight at 80°C) of the mice were recorded periodically to assess the functionality of the GI tract (Puig *et al*. 2012).

### Statistical analysis

All data were analysed using GraphPad Prism 6.0 software (Graphpad Prism, San Diego, CA, USA). Animal weight, food consumption, faecal weight, NOR and Y‐maze tasks were analysed using unpaired two‐tailed Student's *t* tests. Data obtained from the water maze and EFS‐evoked contractile alterations in wave patterns were analysed by two‐way ANOVA followed by Bonferroni multiple comparison tests. *P *< 0.05 was considered to indicate a statistically significant difference between values. Data represent the means ± SD.

## Results

### Spread of Aβ from injections into the body of the stomach and proximal colon within 1 month of intra‐GI administration

**Figure 2:**
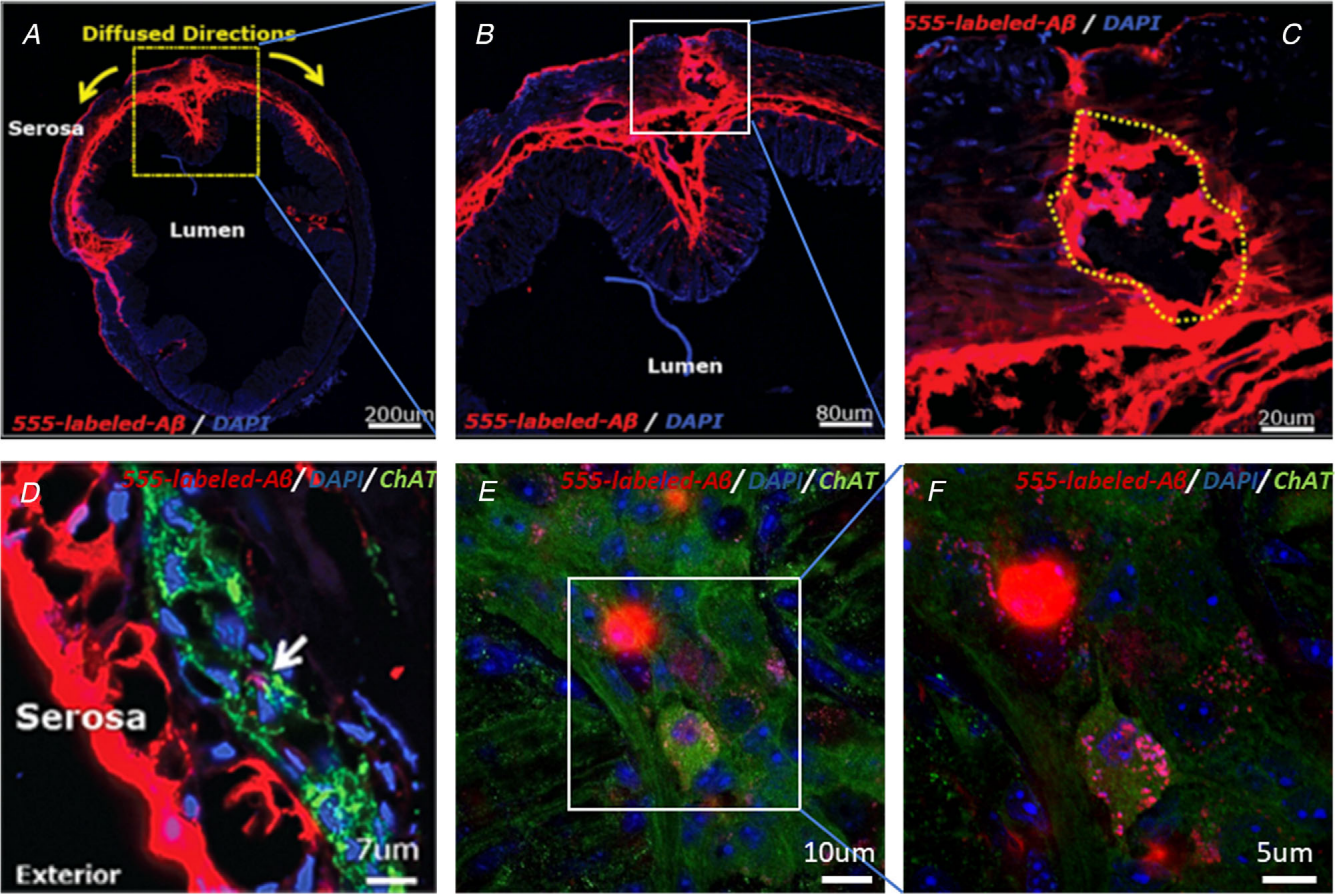
Localisation and distribution of fluorescently labelled Aβ in the proximal colon HiLyte Fluor 555‐labelled Aβ1–42 (shown in red) was detected in the transverse sections of the proximal colon at the original injection sites (yellow‐dotted) by immunofluorescence as described in *Methods*. Aβ was found in the smooth muscle layer and the nearby areas along the submucosa. *Scale bars*: 200 μm, 80 μm and 20 μm (*A–C*). In the transverse sections, fluorescently labelled Aβ was associated with the PGP9.5‐positive neurons of the myenteric plexus (anti‐PGP9.5 is shown in green, and DAPI staining for nuclei is shown in blue). *Scale bar*: 7 μm (*D*). Cholinergic neurons of myenteric plexus were identified and had internalised the HiLyte Fluor 555‐labelled Aβ1–42 (red) 3 h post‐injection, in the whole‐mount staining using DAPI (blue) and anti‐ChAT antibodies (green). *Scale bars*: 10 μm and 5 μm (*E* and *F*). [Color figure can be viewed at wileyonlinelibrary.com]

**Figure 3:**
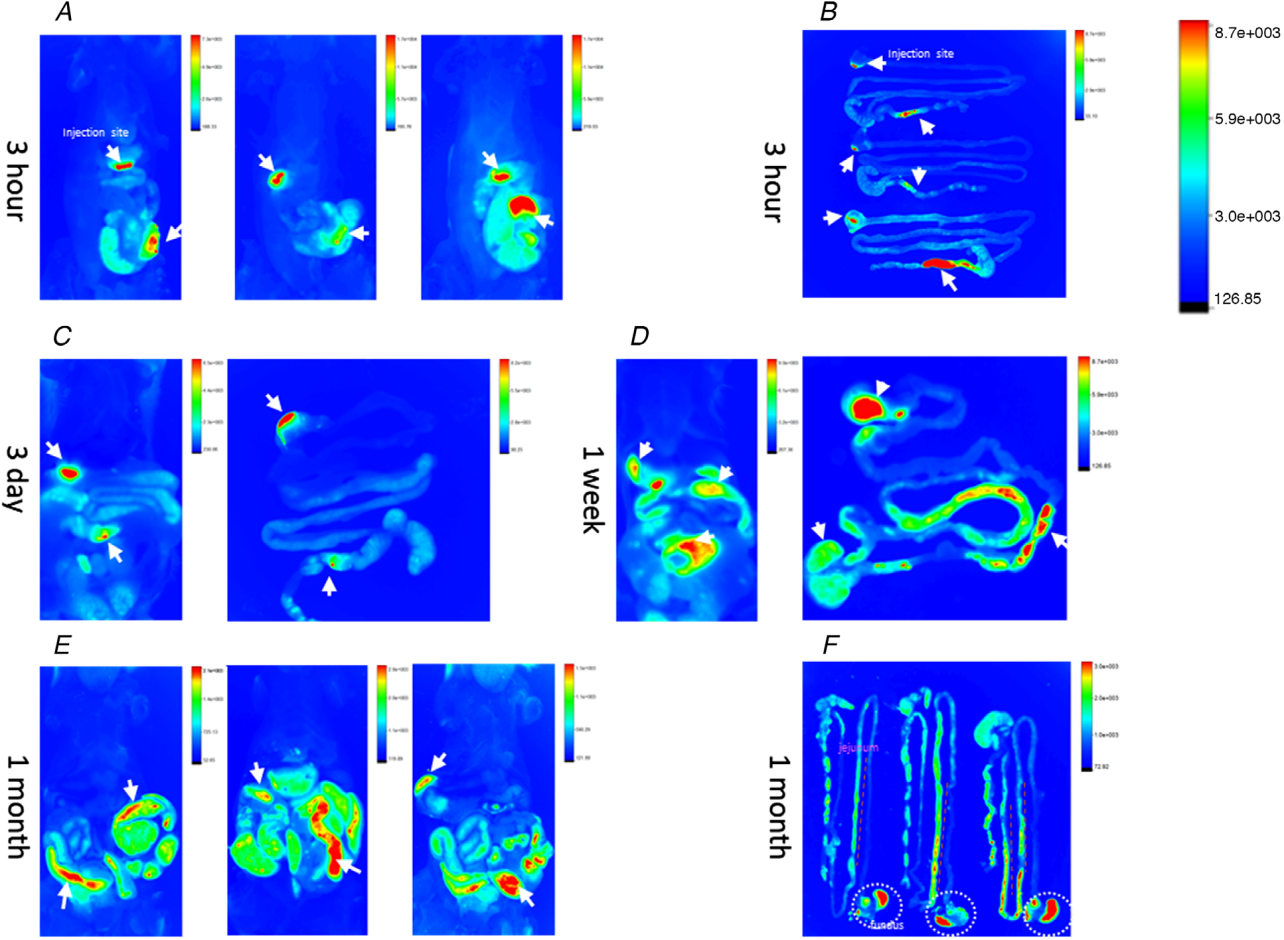
Distributions of HiLyte Fluor 555‐labelled Aβ1–42 in the body of the stomach and proximal colon Images were obtained using the Bruker *in vivo* imaging system. HiLyte Fluor 555‐labelled Aβ1–42 was initially injected into the body of the stomach and proximal colon, and these seeds could be detected 3 h post‐surgery (*A*, *B*). White arrows indicated the site of injection. Fluorescence was still evident in the original areas, without obvious diffusion to other areas of the GI tract 3 days post‐surgery (*C*). After 1 week the oligomers had diffused, and the injected regions, together with the segment of jejunum, displayed highly positive signals (*D*). At 1 month post‐surgery, the signals were detected in the whole stomach and jejunum (*E*, *F*). [Color figure can be viewed at wileyonlinelibrary.com]

To visualise the spread of Aβ from GI injections, the Bruker *in vivo* imaging system was used to identify the fluorescence‐labelled Aβ in the GI tract. Positive signals were detected at transverse sections and myenteric plexuses from segments and examined using confocal laser scanning microscopy (Fig. 2). At 3 h post‐injection, fluorescent signals had diffused to nearby areas (Fig. 2*C*) and had been partially absorbed into cholinergic neurons of the myenteric plexus in the proximal colon (Fig. 2*D*–*F*) and stomach (not shown). At 3 h, *in vivo* imaging revealed that the fluorescent signals were retained in the original sites (the body of the stomach and proximal colon (Fig. 3*A* and *B*), without obvious diffusion to other sites at 3 days (Fig. 3*C*); at 1 week, the signals were not limited to only the original injected areas (Fig. 3*D*). At 1 month, fluorescent signals were present in the fundus and jejunum, together with the originally injected areas (Fig. 3*E* and *F*).

**Figure 4:**
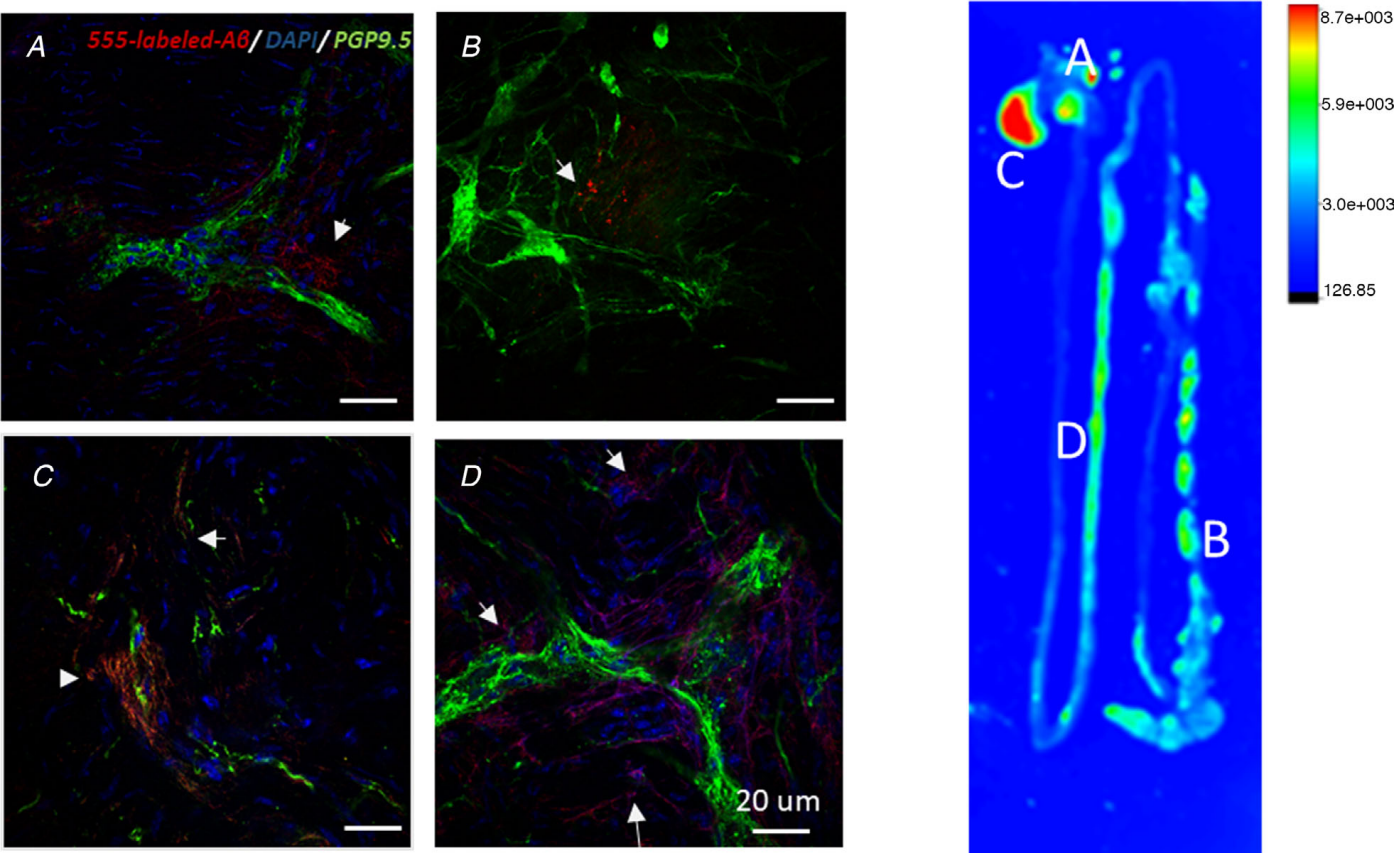
Distributions of HiLyte Fluor 555‐labelled Aβ1–42 in the GI tract after 1 month The distribution of Aβ (red colour) was tracked with confocal laser scanning and anti‐PGP9.5 immunostaining (green colour). Minimal HiLyte Fluor 555‐labelled Aβ1–42 (red colour) were detected close to PGP9.5‐positive neurons in the injected areas of the body of the stomach (*A*) and proximal colon (*B*). The fundus of the stomach (*C*) and the jejunum (*D*) displayed highly positive signals in the first month post‐surgery. White arrows highlighted the spread of HiLyte Fluor 555‐labelled Aβ1–42 signals. *Scale bar*: 20 μm. The distribution of HiLyte Fluor 555‐labelled Aβ1–42 in the GI tract and corresponding area of immunofluorescent image *(Right panel)*. [Color figure can be viewed at wileyonlinelibrary.com]

To visualise the Aβ distribution and neural uptake after 1 month post‐injection, neurons were further stained by anti‐PGP9.5 after dissection. Whole‐mounting staining revealed positive signals in the body of the stomach (Fig. 4*A*) and proximal colon (Fig. 4*B*), fundus (Fig. 4*C*) and jejunum (Fig. 4*D*). A small amount of Aβ consistently remained in the injected areas after 1 month, but mostly diffused to the fundus and jejunum (Fig. 4 right panel).

### Intra‐GI administration of Aβ induces Alzheimer's disease‐like cognitive deficits

**Figure 5:**
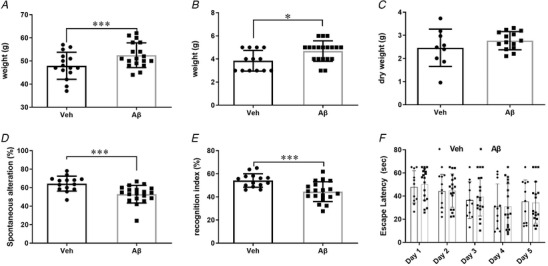
Effects of intra‐GI administration of Aβ or vehicle on cognitive performance Body weight (*A*), daily food intake (*B*) and dry faecal output (*C*). *D*, cognitive performance as displayed in the spontaneous Y‐maze. *E*, novel object recognition test and (*F*) Morris water maze (MWM). Means ± SD are shown. *Group size*: Veh = 15, Aβ = 20. Significant differences between treatment groups are indicated as ^*^ *P *< 0.05, ^***^ *P *< 0.001 (two‐way ANOVA for the MWM task; unpaired Student's *t* test for the other tests).

Three behaviour tests were performed at 1 year post‐administration to evaluate the effect of intra‐GI administered Aβ on cognition. The spontaneous Y‐maze was used to assess the spatial working and short‐term memory. There were no differences in the total number of arm entries between animals in different treatment groups (*P* > 0.05, data not shown) indicating that locomotor activities were comparable. However, the vehicle‐treated animals that had received intra‐GI Aβ exhibited fewer spontaneous alterations (64.34 ± 8.10% *vs* 52.90 ± 9.52%, respectively; Fig. 5*D*, *P *< 0.001), indicating cognitive deficits.

The same mice were assessed for long‐term spontaneous memory by the non‐forced NOR task, two days following the Y‐maze test. By measuring the time percentages of exploring novel objects (recognition indexes), Aβ‐injected mice exhibited significantly less time (%) exploring new objects than the controls, suggesting that the Aβ‐treated mice had impaired long‐term spontaneous memory (Fig. 5*E*, *P *< 0.001).

Finally, the same mice were also tested in the stressful MWM task to assess the spatial learning and flexibility, but there was no significant difference between the treatment groups in either the training session (Fig. 5*F*, *P *> 0.05) or the probe trial test (data not shown).

At the end of 1 year, the Aβ‐treated animals had gained weight, and were 4.6 ± 1.9 g heavier than the vehicle control‐treated animals (Fig. 5*A*; *P *< 0.001). The Aβ‐treated animals also had an increase of 20% in daily food intake (Fig. 5*B*, *P *< 0.05), but there was no increase in dry faecal output (Fig. 5*C*, *P *> 0.05). The results indicate that enteric Aβ increased appetite without affecting the frequency of defecation.

### Intra‐GI administration of Aβ caused cerebral and vagal beta‐amyloidosis

**Figure 6:**
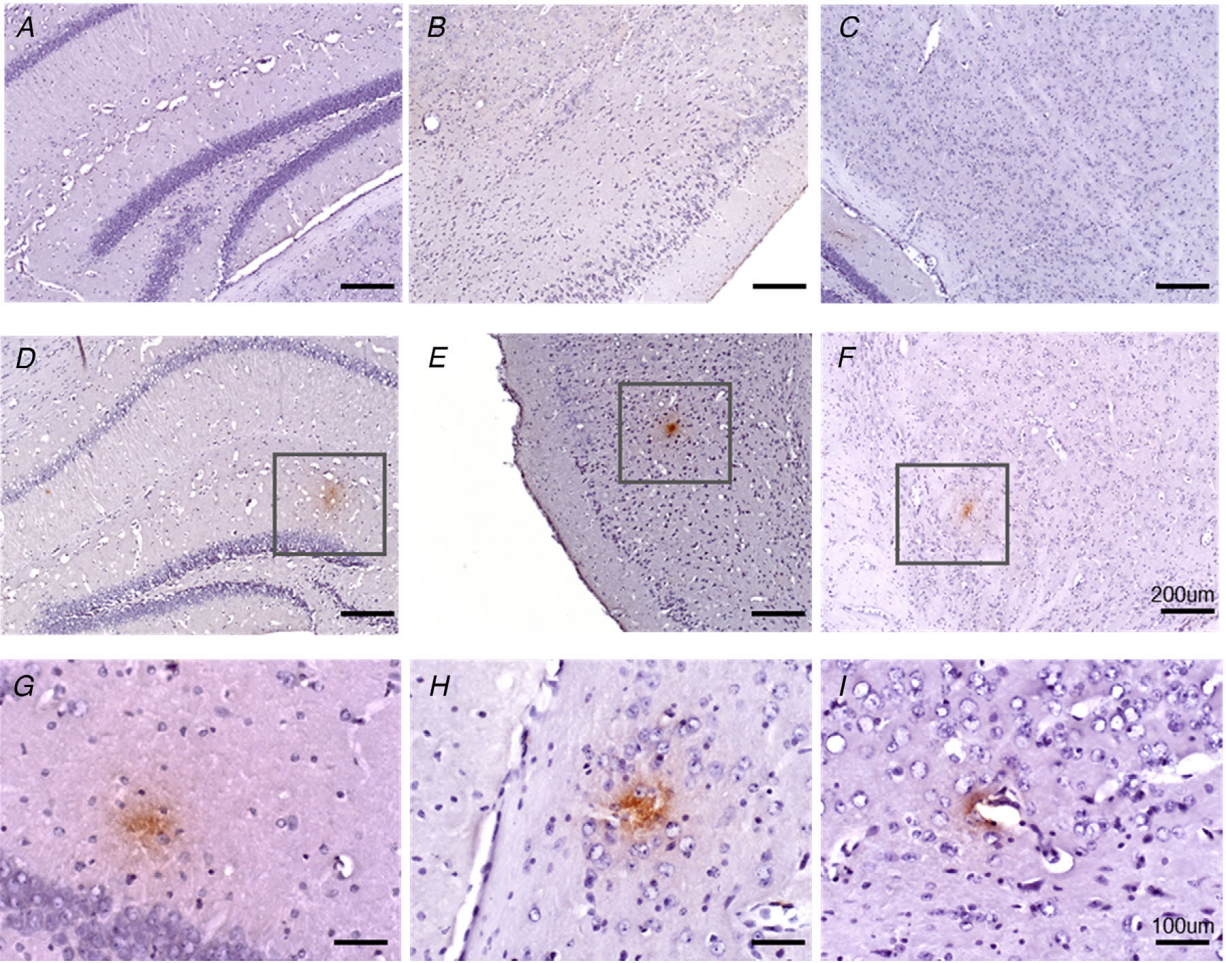
Effects of intra‐GI administration of Aβ or vehicle on the formation of Aβ deposits in the brain The representative images show Aβ immunoreactivity in brain histology sections as described in *Methods*. *A–C*, vehicle (Veh) control group. *D–F*, group receiving Aβ injections in the gut; the frames highlight the Aβ deposits. *Scale bar*: 200 μm. The images in *G–I* also belong to the Aβ group, and *I* depicts the Aβ deposits attached to the vascular walls. *Scale bar*: 100 μm. [Color figure can be viewed at wileyonlinelibrary.com]

**Figure 7:**
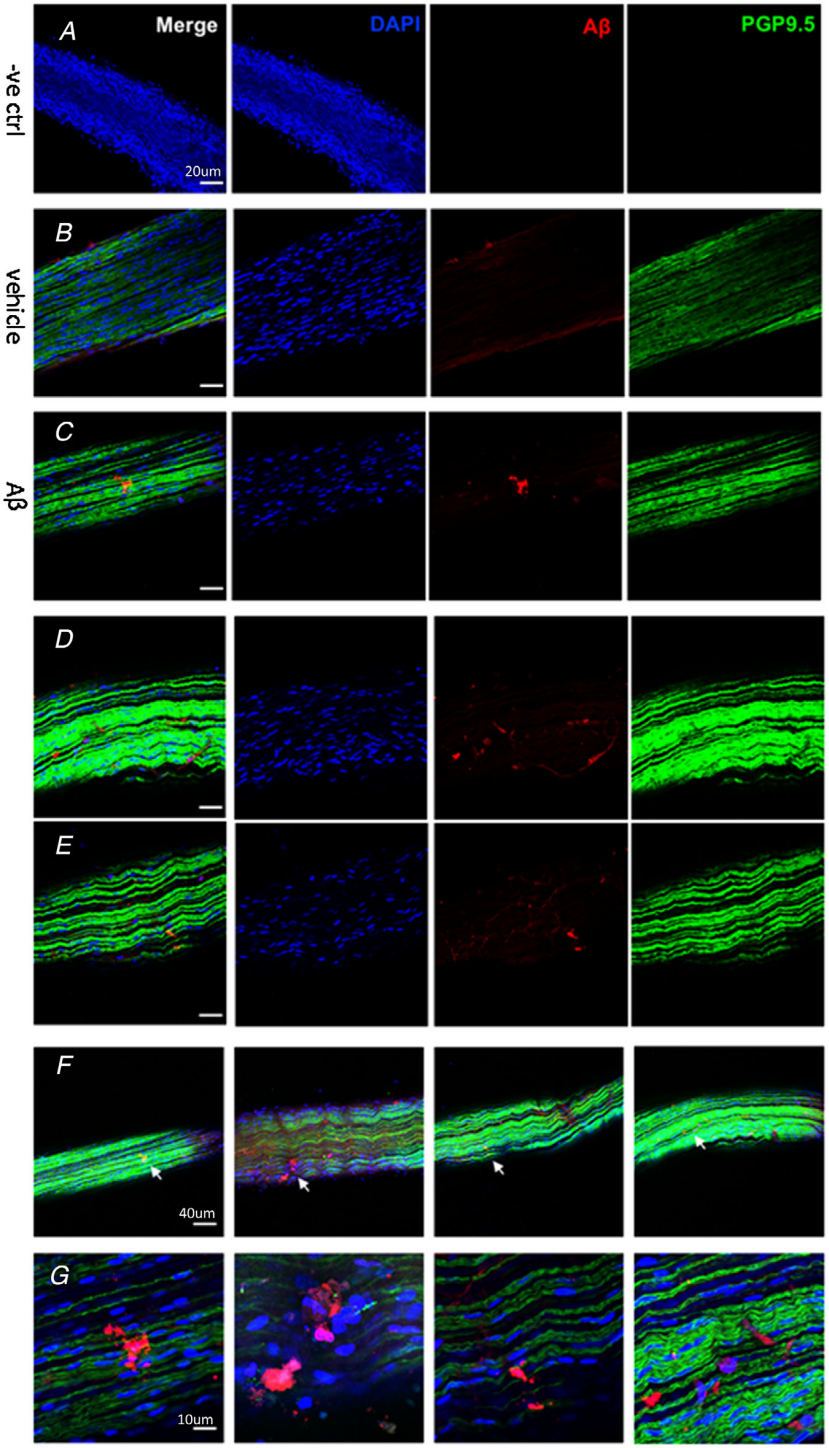
Effects of intra‐GI administration of Aβ or vehicle on the formation of Aβ deposits in the vagus nerve The representative images showed the outcomes of the Aβ immunoreactivity in sections of the vagus nerve. *A*, negative control of Aβ group. *B*, the vehicle (Veh) group. *C–G*, the Aβ group. *Scale bars*: 20 μm (*A–E*), 40 μm (*F*), 10 μm (*G*). [Color figure can be viewed at wileyonlinelibrary.com]

From the reduced cognitive functions, we believed that the intra‐GI administration of Aβ had an effect towards the neural system. The deposition of Aβ plaques were investigated by immunostaining in a series of sections in the brain and vagus nerves. Plaques were widely distributed throughout the whole brains (Fig. 6) and vagi (Fig. 7) of the intra‐GI administered Aβ‐treated animals; plaques were seen in the hippocampus (Fig. 6*D* and *G*), cortex (Fig. 6*F* and *H*), amygdala (Fig. 6*E*) and blood vessel walls (Fig. 6*I*). The staining indicates the uptake of Aβ through the gut–brain axis.

To review the uptake of Aβ in the vagus nerve, the nerves were sectioned and stained for Aβ immunoreactivity. The vagal fibres of Aβ‐treated mice stained positive for Aβ. The morphology of Aβ‐treated vagal fibres had a wavy and tortuous arrangement compared with normal (Fig. 7*C*–*G*). No positive staining was observed in the vehicle‐treated animals (Fig. 6*A*–*C*; Fig. 7*B*).

### Alteration of jejunum activity 1 year after intra‐GI administration of Aβ

**Figure 8:**
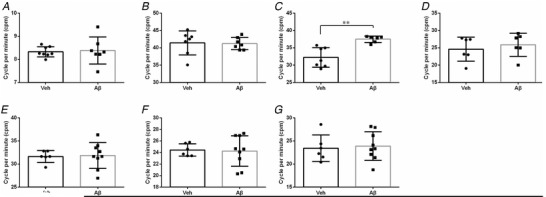
Effects of intra‐GI administration of Aβ or vehicle on gastric spontaneous contraction and slow‐wave alterations in different segments Contractile frequencies of stomach (*A*), duodenum (*B*), jejunum (*C*) and ileum (*D*) were recorded *ex vivo* via organ bath. Cycle rate per minute (cpm) was defined to represent dominant frequencies of slow waves in the duodenum (*E*), jejunum (*F*) and ileum (*G*). Data are shown as means ± SD. *Group size*: *A*, Veh = 7, Aβ = 8; *B*, Veh = 7, Aβ = 7; *C*, Veh = 6, Aβ = 7; *D*, Veh = 6, Aβ = 6; *E*, Veh = 6, Aβ = 9; *F*, Veh = 6, Aβ = 9; *G*, Veh = 6, Aβ = 9. Significant differences between treatment groups are indicated as ^*^ *P *< 0.05, ^**^ *P *< 0.01 (unpaired Student's *t* test).

The spontaneous contractions, intestinal slow waves and GI neuronal coupling deficits were measured at 1 year post‐surgery. Aβ treatment had enhanced the spontaneous contractile response cycle rate of jejunum tissues to 37.5 ± 0.9 cpm from 32.2 ± 2.8 cpm (Fig. 8*C*; *P *< 0.01). Aβ treatment had no effect on the contractile frequency of tissues from the stomach, duodenum and ileum (Fig. 8*A*, *B* and *D*; *P* > 0.05).

**Figure 9:**
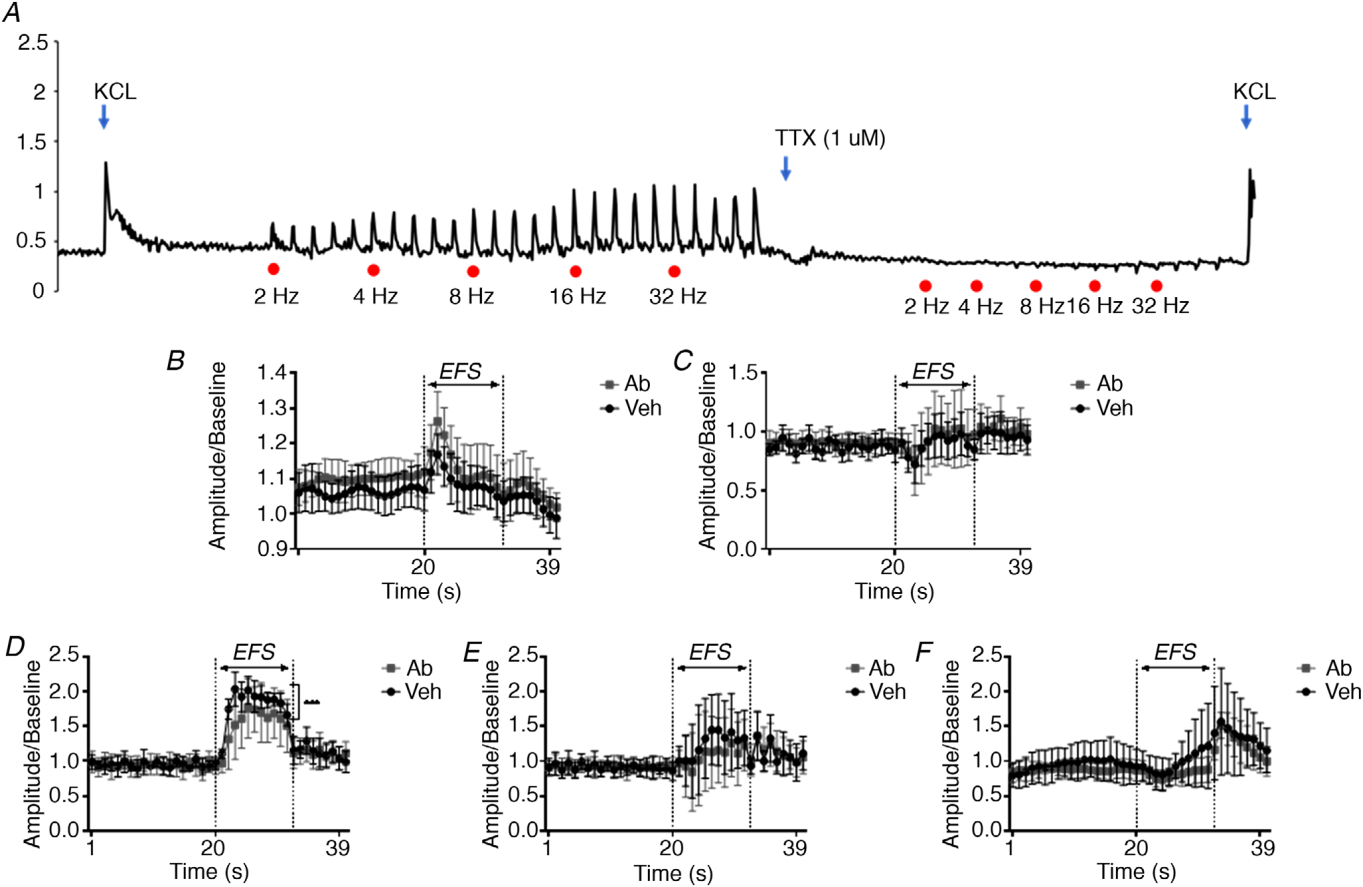
Effects of enteric administration of Aβ on neuronal coupling deficits At basal tension, EFS induced contractile responses the amplitude of which increased with increasing stimulation frequency. These responses triggered by EFS (at 2, 4, 8, 16 and 32 Hz, red dots) were dramatically suppressed or even abolished by 1 μM TTX, indicating their neuronal nature as a previous study described (*A*). EFS‐induced wave patterns of stomach (*B*), duodenum (*C*), jejunum (*D*), ileum (*E*) and proximal colon (*F*) were collected and normalised as described in the *Methods* section. For instance, 40 s records were selected to draw the wave patterns of each segment, and raw data were normalised by the last 20 s baseline prior to EFS (at 8 Hz). To clarify different contributions of enteric Aβ to the wave pattern alterations, data were selected for the comparison. Data represent the means ± SD. Significant differences between treatment groups are calculated as ^*^ *P *< 0.05, ^**^ *P *< 0.01, ^***^ *P *< 0.001 (two‐way ANOVA followed by Bonferroni multiple comparison tests). [Color figure can be viewed at wileyonlinelibrary.com]

EFS was used to assess neuromuscular coupling sensitivity of the tissue segments. All the electrically elicited motor responses of longitudinal muscle rings were suppressed or abolished in the presence of 1 μm TTX (a blocker of neuronal conductance) (Fig. 9*A*), indicating that the wave patterns observed were of an intrinsic nerve origin. Among a series of frequencies examined, 8 Hz was selected as a typical parameter to probe the neuronal dysfunction. After normalising the raw data to baseline, we constructed an EFS‐induced wave pattern for each segment (Fig. 9*B–F*). In the stomach segments, EFS generally induced an immediate EFS‐on contraction during stimulation. The segments from different groups shared the same wave pattern, and Aβ treatment did not induce alteration of the contractile amplitudes during the EFS‐on phase, which could be subsequently blocked by TTX (Fig. 9*B*). The duodenum had a different pattern: an EFS‐on relaxation, which was not different between the two groups (Fig. 9*C*, *P *> 0.05).

In the jejunum, the EFS‐induced contractions in the Aβ‐treated animals had suppressed amplitude, compared with the vehicle controls (Fig. 9*D*, *P* < 0.001). Conversely, the ileum demonstrated no difference in either wave pattern or contractile response between the vehicle and Aβ groups (Fig. 9*E*, *P *> 0.05).

Finally, the recordings of proximal colon display an EFS‐off contraction in response to EFS, quite different from the other segments. However, no differences existed between the tissues obtained from the vehicle‐ and Aβ‐treated animals (Fig. 9*F*, *P *> 0.05).

## Discussion

### Impaired cognitive function in the GI‐driven dementia model

In the present study, we established a new GI‐driven dementia model in the mouse. A series of memory tests revealed that intra‐GI tract injection of Αβ oligomers had detrimental effects on the cognitive capability of mice 1 year post‐injection. The spontaneous Y‐maze and NOR tasks demonstrated both short‐ and long‐term spatial memory deficits. The results were comparable to an established intracerebroventricular Αβ injection model (Kim *et al*. 2016), confirming the effect of GI Αβ injection.

The MWM task did not efficiently distinguish a potential difference in cognition. The possible explanations could be that: (1) the memory impairments of the GI model might not result from hippocampal degeneration, as the MWM task depends heavily upon the hippocampus; (2) because of the stress of the MWM task itself, subjects might respond individually or differently in the memory acquisition session (Irvine *et al*. 2005). Other murine dementia models reported insignificant differences (Faucher *et al*. 2016; Wolf *et al*. 2016). A review of 10 murine dementia models highlighted that variability between testing laboratories may involve differences in age, strain, type of peptide, and/or testing protocols/tasks (Webster *et al*. 2014).

### Αβ effect on the gut and metabolism

The enteric Αβ seeds did not appear to adversely affect long‐term GI functioning in terms of effects on gastric slow waves, produced by interstitial cells of Cajal, when analysed at 1 year. However, there was an alteration of functioning in terms of spontaneous contractions and neuronal couplings of the jejunum, which did not result in constipation, as demonstrated by the lack of changes in dry or wet weight of faecal output. Another dementia model used by Semar *et al*.(2013), suggested a weaker but higher frequency contraction pattern in the AβPP transgenic mouse. Weight gains were observed in our model, and the transgenic Tg4‐42 model is also heavier than wild‐type controls (Wagner *et al*. 2019), while a transgenic BxFAD dementia mouse model may have weight loss (Brandscheid *et al*. 2017). Both positive or negative weight changes have been associated with dementia in humans depending on their disease progression (Ikeda *et al*. 2002).

Nevertheless, AD patients are not known to have constipation or overt gastric dysfunction prior to cognitive deficits, unlike PD patients (Toru *et al*. 2018). PD is characterised by a range of non‐motor symptoms preceding the motor phase (Poewe 2008), and GI dysfunction is considered as one of the most common non‐motor symptoms of PD.

### Αβ delivery to brain via the vagus nerve

To identify whether the intra‐GI tract injection of Αβ oligomers could trigger misfolded Αβ deposits in the brain via the vagus nerve, we performed immunohistochemical and immunofluorescence staining. Aggregation was consistently detected in the areas of the hippocampus and in other brain regions. Among the widely distributed cerebral amyloidosis, we also observed some deposits attached to the vessel walls, typically consistent with the syndrome of cerebral amyloid angiopathy (CAA), which could result in degeneration of the vessel walls and haemorrhages. Although clinically insufficient to be a diagnostic criterion, CAA is present in nearly 80% of AD patients (Biffi & Greenberg 2011). Additionally, we observed Αβ deposits in the vagus with loosely arranged vagal fibres, consolidating our hypothesis that axonal transportation via vagal nerves is one available route to facilitate migration of enteric Αβ to the brain, much as in prion and Parkinson's diseases (Visanji *et al*. 2013; Kujawska & Jodynis‐Liebert 2018).

From our results, the enteric Αβ seeds remained in the GI tract for at least 1 month and had been taken up into cholinergic neurons of the myenteric plexus. The internalisation into neurons in the enteric nervous system appears to be in accordance with the known high affinity of Αβ to become internalised into cholinergic neurons in the brain of AD patients (Kar *et al*. 2004; Schliebs and Arendt 2011). Our data indicate a potential peripheral mechanism of Αβ to gain access to the enteric nervous system whereby it may further translocate to the CNS via axons in the vagal nerves.

Other data revealed the role of prion propagations in neurodegenerative diseases (Goedert *et al*. 2010), of which AD and PD shared multiple mechanisms (Xie *et al*. 2014). Recently, it has been shown that the protein intimately involved in PD, α‐synuclein, could retrogradely move from the gut to the brain (Braak *et al*. 2006; Probst, Bloch, and Tolnay 2008; Lebouvier *et al*. 2009; Holmqvist *et al*. 2014). Comparatively, Αβ deposits have already been observed in the GI tract of AD patients and also in transgenic mice overexpressing APP (Eisele *et al*. 2007; Eisele & Duyckaerts 2016); the intraperitoneal injection of Αβ enhanced the aggregation of Αβ in the brain of transgenic mice, an observation that generally supports the retrograde transport route. Schwiertz *et al*., found an increased intestinal permeability in PD (Schwiertz *et al*. 2018), which can be a potential mechanism to promote protein(s) passing the gut wall and propagation. However, direct evidence has been lacking to demonstrate that enteric Αβ seeds can retrogradely invade the CNS to induce AD symptoms.

To our knowledge, we are the first to demonstrate that an intra‐GI administration of Aβ oligomers could directly induce Aβ assemblies in the brain and cause memory impairments. However, there seem to be alternative access pathways for the enteric Aβ seeds to invade the brain and cause dementia, such as retrograde axonal transport through the vagal nerves and haematogenous routes, as seen in prion pathology (Watts *et al*. 2014). Plenty of experimental evidence supports the existence of axonal transport in the pathology of AD, as Αβ deposits initially occur in the neocortex and hippocampus, followed by the lenticular nucleus, thalamus, mesenphalon and finally the cerebellum of AD patients (Jarrett & Lansbury Jr. 1993; Jarrett *et al*. 1993). Moreover, Braak's hypothesis, which is based on PD, emphasised that the pathogen α‐synuclein could be transported retrogradely via vagal nerves, and vagotomy has been shown to eliminate the transport from the gut to the CNS (Svensson *et al*. 2015a, 2015b). Remarkably, intravenously administered α‐synuclein led to deposits in the brain, suggesting that it could also be transported via blood across the blood–brain barrier (Peelaerts *et al*. 2015). In fact, Αβ may have enhanced access to the CNS in CAA via unidentified mechanisms. Recently, a study also revealed that circulating monocytes are vectors to deliver Αβ seeds from the periphery to the brain (Cintron *et al*. 2015).

All this evidence sheds light on the potential contributory mechanisms of retrograde axonal and/or immune transport of Αβ from the periphery to brain, with central degeneration occurring in a prion‐like manner. The underlying mechanism for how the enteric Αβ induced neuro‐invasion of the CNS is unclear. The GI tract is a window to the environment and in susceptible individuals may be the starting point of Αβ toxicity. Together with the present observations, our results are to our knowledge the first to demonstrate that enteric Αβ administration directly induces AD‐like dementia and cerebral amyloidosis, which may be induced by the retrograde axonal transportation via the vagus. If the characteristic alterations initially occur in the GI tract ahead of the brain, it may open the possibility of designing therapies to delay or prevent the translocation of Αβ, to protect the CNS from unwanted neurodegenerative changes.

## Additional information

### Competing interests

The authors declare that they have no conflicts of interest.

### Author contributions

YS and JAR designed the study. YS carried out the experiments, analysed the data and wrote the draft manuscript. NRS assisted in establishing the animal models. YL participated in the data analysis of MEA. MN assisted in the behavioural recordings. DP contributed to the organ bath experiments. ZL and JSCK interpreted the data. YS, DP, ZL, JSCK and JAR prepared the manuscript. All authors read and approved the final manuscript.

## Supporting information


**Statistical Summary Document**

## Data Availability

Data are available on request from the authors.
