# Supplementary material for: Intra‐gastrointestinal amyloid‐β1–42 oligomers perturb enteric function and induce Alzheimer's disease pathology
Source: J Physiol. 2020 Jul 6;598(19):4209–23. doi: 10.1113/JP279919 (PMC7586845; doi:10.1113/JP279919)
Supplement: Supplementary file 1 — Statistical Summary Document [file TJP-598-4209-s001.docx]

**Manuscript Title:** Intra-Gastrointestinal Amyloid-β1-42 Oligomers Perturb Enteric Function and Induce Alzheimer’s Disease Pathology

**Authors:** Yayi Sun, Nerina R. Sommerville, Julia Yuen Hang Liu, Man Piu Ngan, Daniel Poon, Eugene D. Ponomarev, Zengbing Lu, Jeng S. C. Kung, John A. Rudd

**Animal model used, if applicable:** Mouse – ICR

**Underlying hypothesis:** This investigation tests the hypothesis that gastrointestinal (GI) tract injection of beta-amyloid (Aβ) induces Alzheimer’s Disease pathology, compare to vehicle control.

**Definitions of ‘n’:**

Question 1: n = number of animals from which multiple tissue sections were stained and observed.

Question 2: n = number of samples from different animals for staining

Question 3: n = number of animals in vehicle/treatment group

Question 4: n = number of animals in vehicle/treatment group

Question 5: n = number of animals from which multiple tissue sections were stained and observed

Question 6: n = number of animals from which multiple tissue sections were stained and observed

Question 7: n = number of samples from different animals of the vehicle/treatment group

Question 8: n = number of data points of time-series data

**Statistical summary table:**

| Experimental question number* | Finding/ conclusion | Experimental location/ variable  e.g. muscle, neocortex or genotype | Mean value  (or other summary statistic) | SD | n val. | P** | Units | Data comparisons  e.g. WT vs KO | Statistical test | Any other variable  e.g. subjects’ age or sex | Figure/ table in which data are presented | Comments  e.g. observation |
| --- | --- | --- | --- | --- | --- | --- | --- | --- | --- | --- | --- | --- |
| 1. Location of Aβ and spread? | Confirmed by immunofluorescent staining | GI tract | - | - | 2 | - | - | Aβ | Observation | - | 2 | Positive staining of 555-labeled-Aβ at GI tract serosa |
|  | Confirmed by in-vivo imaging | Abdomen | - | - | 8 | - | - |  |  | - | 3 | Strong signal of 555-labeled-Aβ along injection sites |
| 2. Neural uptake of Aβ? | Confirmed by IF | GI tract | - | - | 2 | - | - | Aβ | Observation | - | 4 | Signals of 555-labeled-Aβ were found along PGP9.5 staining of neurons |
| 3. Difference in | Higher in Aβ | Aβ | 52.50 | 5.36 | 20 | - | g | Aβ vs vehicle | Students’ t test, unpaired | - | 5A | 95% CI [0.5873 – 8.546] |
| Weight? |  | Vehicle | 47.93 | 5.83 | 15 | **0.026** |  |  |  | - |  |  |
|  | Higher in Aβ | Aβ | 4.68 | 0.89 | 19 | **-** | g |  |  | - | 5B | 95% CI [0.1834-1.493] |
| Appetite? |  | Vehicle | 3.85 | 0.90 | 13 | **0.014** |  |  |  | - |  |  |
|  | No difference | Aβ | 2.77 | 0.39 | 14 | - | g |  |  | - | 5C | - |
| Constipation? |  | Vehicle | 2.46 | 0.81 | 9 | 0.234 |  |  |  | - |  |  |
| 4. Alter cognitive performance? | Reduce spontaneous alterations | Aβ | 52.90 | 9.52 | 19 | - | % | Aβ vs vehicle | Students’ t test, unpaired  Students’ t test, unpaired | - | 5D | 95% CI [-17.87- -5.021] |
|  |  | Vehicle | 64.34 | 8.075 | 14 | **0.001** |  |  |  | - |  |  |
|  | Reduce recognition index | Aβ | 44.51 | 8.577 | 19 | - | % |  |  | - | 5E | 95% CI [-15.12- -4.257] |
|  |  | Vehicle | 54.20 | 5.8564 | 14 | **0.001** |  |  |  | - |  |  |
|  | No difference in Morris water maze test | Aβ | 40.03 | 7.701 | 5 | - | second |  | Two-way ANOVA | - | 5F | - |
|  |  | Vehicle | 38.99 | 6.920 | 5 | 0.935 |  |  |  | - |  |  |
| 5. Any Aβ deposits in brain? | Yes, by anti-Aβ IHC | Brain | - | - | 6 | - | - | Aβ vs vehicle | Observation | - | 6 | Aβ plaques observed in brain sections |
| 6. Any Aβ deposits in the vagus nerve? | Yes, by presence of 555-labeled-Aβ | Vagus nerve | - | - | 6 | - | - | Aβ vs vehicle | Observation | - | 7 | Aβ plaques observed with nerve fibres, with distorted fibres arrangement. |
| 7. Difference in gastric spontaneous contractions? | No difference in Stomach | Stomach: Aβ | 8.383 | 0.5823 | 7 | - | cpm | Aβ vs vehicle | Students’ t test, unpaired | - | 8A | - |
|  |  | Vehicle | 8.329 | 0.2210 | 8 | 0.810 |  |  |  | - |  |  |
|  | No difference in Duodenum | Duodenum: Aβ | 41.21 | 1.739 | 7 | - |  |  |  | - | 8B | - |
|  |  | Vehicle | 41.41 | 3.476 | 7 | 0.894 |  |  |  | - |  |  |
|  | Reduction in Jejunum contractile frequencies | Jejunum: Aβ | 37.48 | 0.9304 | 6 | - |  |  |  | - | 8C | 95% CI [2.595-7.951] |
|  |  | Vehicle | 32.21 | 2.837 | 7 | **0.001** |  |  |  | - |  |  |
|  | No difference in Ileum | Ileum: Aβ | 25.87 | 3.349 | 6 | - |  |  |  | - | 8D | - |
|  |  | Vehicle | 24.62 | 3.477 | 6 | 0.538 |  |  |  | - |  |  |
| slow waves? | No difference in jejunum | Aβ | 24.26 | 2.636 | 9 | - | cpm | Aβ vs vehicle | Students’ t test, unpaired | - | 8E | - |
|  |  | Vehicle | 24.44 | 1.068 | 6 | 0.879 |  |  |  | - |  |  |
|  | No difference in duodenum | Aβ | 31.86 | 2.793 | 9 | - |  |  |  | - | 8F | - |
|  |  | Vehicle | 31.62 | 1.284 | 6 | 0.851 |  |  |  | - |  |  |
|  | No difference in ileum | Aβ | 23.89 | 3.087 | 9 | - |  |  |  | - | 8G | - |
|  |  | Vehicle | 23.43 | 2.876 | 6 | 0.777 |  |  |  | - |  |  |
| 8. Any neural coupling deficits? | No difference in stomach | Stomach: Aβ | 1.101 | 0.044 | 40 |  | Proportion, amplitude/baseline | Aβ vs vehicle | two-way ANOVA followed by Bonferroni multiple comparison tests | - | 9B | - |
|  |  | Vehicle | 1.064 | 0.031 | 40 | 0.859 |  |  |  |  |  |  |
|  | No difference in duodenum | Duodenum: Aβ | 0.9405 | 0.069 | 40 |  |  |  |  |  | 9C |  |
|  |  | Vehicle | 0.9001 | 0.064 | 40 | 0.998 |  |  |  |  |  |  |
|  | Reduced amplitude in Aβ group | Jejunum: Aβ | 1.148 | 0.2840 | 40 |  |  |  |  |  | 9D | Significant difference at time point 22 & 23. |
|  |  | Vehicle | 1.226 | 0.3968 | 40 | **<0.001** |  |  |  |  |  |  |
|  | No difference in ileum | Ileum: Aβ | 1.010 | 0.1256 | 40 |  |  |  |  |  | 9E | - |
|  |  | Vehicle | 1.049 | 0.1871 | 40 | 0.951 |  |  |  |  |  |  |
|  | No difference in colon | Colon: Aβ | 0.9410 | 0.1834 | 40 |  |  |  |  |  | 9F |  |
|  |  | Vehicle | 1.047 | 0.1983 | 40 | 0.999 |  |  |  |  |  |  |

*You may use multiple lines for the same question to indicate multiple comparisons

** Authors may wish to make the text bold where p is considered significant against a stated confidence limit.
